# Supplementary material for: PhageMind: generalized strain-level phage host range prediction via meta-learning
Source: Bioinformatics. 2026 Jul 7;42(Suppl 1):btag262. doi: 10.1093/bioinformatics/btag262 (PMC13340165; doi:10.1093/bioinformatics/btag262)
Supplement: btag262_Supplementary_Data [file btag262_supplementary_data.pdf]

PhageMind: Supplemental Document

1. SUPPLEMENTARY NOTE 1: DETAILED FEATURE EXTRACTION PROTOCOLS

S1.1 Bacterial O-antigen Identification Strategy

Microbial O-antigen biosynthesis gene clusters vary significantly in size (5 to 18 genes [1]) due to the complexity of genus-specific glycosyl repeat units. To achieve strong feature generalization, we utilized conserved housekeeping genes as genomic anchors.

For well-characterized genera like *Escherichia* and *Klebsiella*, we extracted the entire polysaccharide synthesis region located between specific housekeeping pairs (Table S1). For *Vibrio*, we adapted the anchoring strategy to reflect species-specific genomic contexts. For *Alteromonas*, where prior knowledge is limited, we employed a cross-generic homology mapping process. O-antigen proteins from model organisms were clustered using MMseqs2 [2] to create a homology library, and target strain contigs were aligned using RagTag [3] to reconstruct gene adjacency and verify flanking housekeeping genes.

Table S1. Housekeeping genes used as genomic anchors for O-antigen cluster identification.

Genera/Species	Housekeeping Gene Anchors
Escherichia [4, 5]	*galF – gnd – hisI*
Klebsiella [6]	*galF – gnd – hisI*
Vibrio metschnikovii [7]	*gmhD – rjg*
Vibrio cholerae [8]	*gmhD – rjg*
Vibrio vulnificus	*gmhD – rjg*
Vibrio alginolyticus [9]	*gmhD – rjg*
Vibrio splendidus [10]	*gmhD – gpm*
Vibrio harveyi [11]	*lpxM – kdkA*
Vibrio parahaemolyticus [12]	*gmhD – dgkA*

Gene Key: *galF*: UTP–glucose-1-phosphate uridylyltransferase; *gnd*: 6-phosphogluconate dehydrogenase; *hisI*: phosphoribosyl-AMP cyclohydrolase; *gmhD*: ADP-L-glycero-D-manno-heptose-6-epimerase; *rjg*: right junction gene (metallo-beta-lactamases); *gpm*: phosphoglyceromutase (2,3-bisphosphoglycerate-independent phosphoglycerate mutase); *lpxM*: lauroyl-Kdo(2)-lipid IV(A) myristoyltransferase; *kdkA*: 3-deoxy-D-manno-octulosonic acid kinase; *dgkA*: diacylglycerol kinase alpha.

S1.2 Phage Tail Fiber Identification

Tail fiber proteins were identified using a combination of automated and manual methods. Initial functional annotation was performed using Pharokka [13]. Potential coding regions were identified based on adjacency to tail assembly genes. To ensure accuracy, candidates underwent manual validation and 3D structural analysis using AlphaFold2 [14] to confirm domain architecture.

S1.3 Numerical Feature Engineering

Both protein sequences and their corresponding DNA sequences were transformed into numerical features (Table S2) [15]. To unify dimensions across varying cluster sizes:

- **Bacteria:** Quartile-based statistics (mean, std, min, max, q25, q50, q75) were computed for the set of O-antigen proteins.
- **Phages:** Simplified statistics (mean, min, max) were computed for the tail fiber proteins.

Table S2. Physicochemical and sequence features extracted for Deep Learning input.

Sequence Type	Feature Category	Dimensions
Protein	Amino acid frequency	20
	Z-scale	5
	CTDC (Composition) [16]	39
	CTDT (Transition) [17]	39
	**Physicochemical properties (16 total):**	
	Isoelectric point (pI)	1
	Aromaticity, flexibility, instability	3
	Fraction of alcohol, aliphatic, amide	3
	Fraction of hydrophobic, sulfur	2
	Fraction of positive, negative charge	2
	Fraction of polar, nonpolar	2
	Structure of helix, sheet, turn	3
DNA	Base frequency	4
	GC content	1
	Codon frequency	64
	Codon usage bias	64

2. SUPPLEMENTARY NOTE 2: DETAILED META-LEARNING FRAMEWORK

S2.1 Loss function

To train the meta-learning framework, we adopted a focal loss function to better handle class imbalance between positive and negative phage–bacterium interactions. In our implementation, the weighting between positive and negative samples is automatically computed based on the number of interaction pairs entering the loss at each iteration. This ensures that the contribution of each class is dynamically adjusted, preventing domination by the majority class and improving stability during training. The standard focal loss is defined as:

$$\mathrm{Focal}(p_t) = -\alpha_t \cdot (1 - p_t)^{\gamma} \cdot \log(p_t) \tag{S1}$$

where p_t is the predicted probability for the true class, α_t is the class-balancing factor, γ is the focusing parameter that reduces the relative loss for well-classified examples and emphasizes harder, misclassified ones (in our case we use $\gamma = 4$). In practice, this formulation allows the model to focus more on challenging phage–host pairs while maintaining balanced contributions from positive and negative interactions.

S2.2 Network Architecture Details

To ensure consistent feature dimensions before entering the GCN, bacterial and phage inputs are first processed through separate heterogeneous FC layers (as stated in Section 2.3.2). This step maps the bacterial features (originally 1764 dimensions) and phage features (originally 756 dimensions) into a unified 64-dimensional space.

$$H_B = \mathrm{ReLU}(W_B \times X_B), \quad H_P = \mathrm{ReLU}(W_P \times X_P) \tag{S2}$$

where $X_B \in \mathbb{R}^{1764 \times n_B}$ and $X_P \in \mathbb{R}^{756 \times n_P}$ are input matrices, $W_B \in \mathbb{R}^{64 \times 1764}$ and $W_P \in \mathbb{R}^{64 \times 756}$ are the weights.

Next, an adaptor module reduces the dimensionality from 64 to 16, then projects back from

16 to 64. The output of this adaptor is added to the original 64-dimensional representation, yielding the final 64-dimensional node features used by the GCN. This helps compress information into a smaller space and then re-express it, while the addition acts like a residual connection to keep the original signals intact. The adaptor improves feature quality and stability.

$$H'_B = H_B + W'_{aB} \times \mathrm{ReLU}(W_{aB} \times H_B), \quad H'_P = H_P + W'_{aP} \times \mathrm{ReLU}(W_{aP} \times H_P) \tag{S3}$$

where $H_B \in \mathbb{R}^{64 \times n_B}$ and $H_P \in \mathbb{R}^{64 \times n_P}$ are outputs from heterogeneous FC layers, $W_{aB} \in \mathbb{R}^{16 \times 64}$, $W'_{aB} \in \mathbb{R}^{64 \times 16}$, $W_{aP} \in \mathbb{R}^{16 \times 64}$, and $W'_{aP} \in \mathbb{R}^{64 \times 16}$ are the weights.

Then we utilize a two-layer GCN architecture which consists of two standard graph convolutional layers, which progressively reduce the dimensionality of the node features from 64, 32 to 16. This gradual reduction allows the network to capture higher-order structural information while compressing the representation into a more compact form.

$$H^{(l+1)} = \mathrm{ReLU}(\tilde{D}^{-\frac{1}{2}} \tilde{A} \tilde{D}^{-\frac{1}{2}} H^{(l)} W^{(l)}) \tag{S4}$$

Following the GCN, an additional FC layer reduces the representation to 8 dimensions. For prediction, the bacterial and phage embeddings are combined by element-wise addition, producing a joint representation of the pair. This combined vector is then passed to the final scoring FC layer, which outputs the predicted interaction probability.

Activation functions are applied consistently throughout the network:

1. ReLU is used in all intermediate layers (heterogeneous FC, adaptor, GCN, and FC).
2. Sigmoid is used in the final scoring layer to map the output to a probability between 0 and 1.

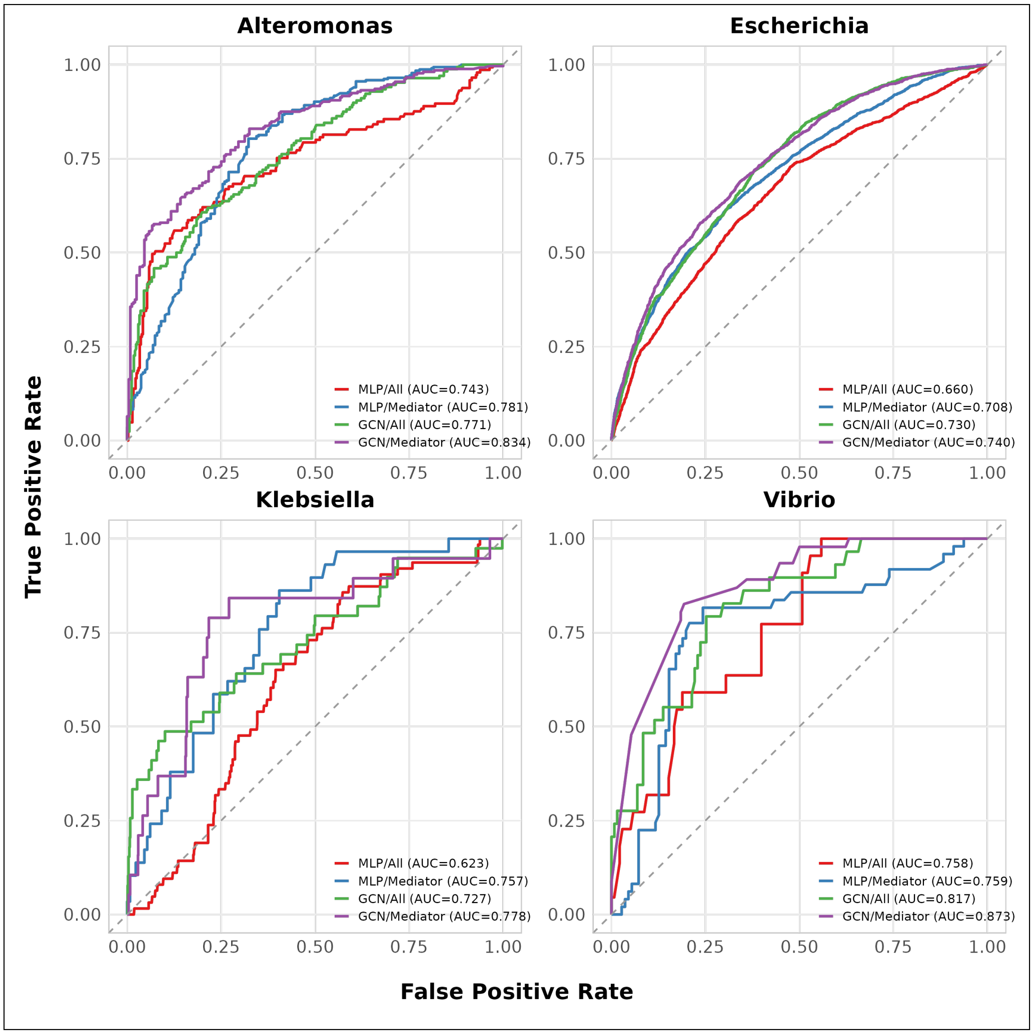

Fig. S1. ROC curves comparing feature and model combinations across four genera. Each panel shows the receiver operating characteristic for one genus, with four curves corresponding to the model–feature combinations. Mediator: using features from O-antigen proteins in bacterial side and tail fiber proteins in phage side; All: using features from all proteins in both side. The diagonal dashed line denotes random performance. Curves closer to the top-left indicate better discrimination. Across all genera, both using the GCN architecture and restricting inputs to mediator features (O-antigen for bacteria; tail fibers for phages) improve classification performance relative to MLP and/or all protein features. The combination GCN/Mediator consistently yields the best ROC behavior, achieving the highest true positive rates at low false positive rates and the most favorable trade-off between sensitivity and specificity.

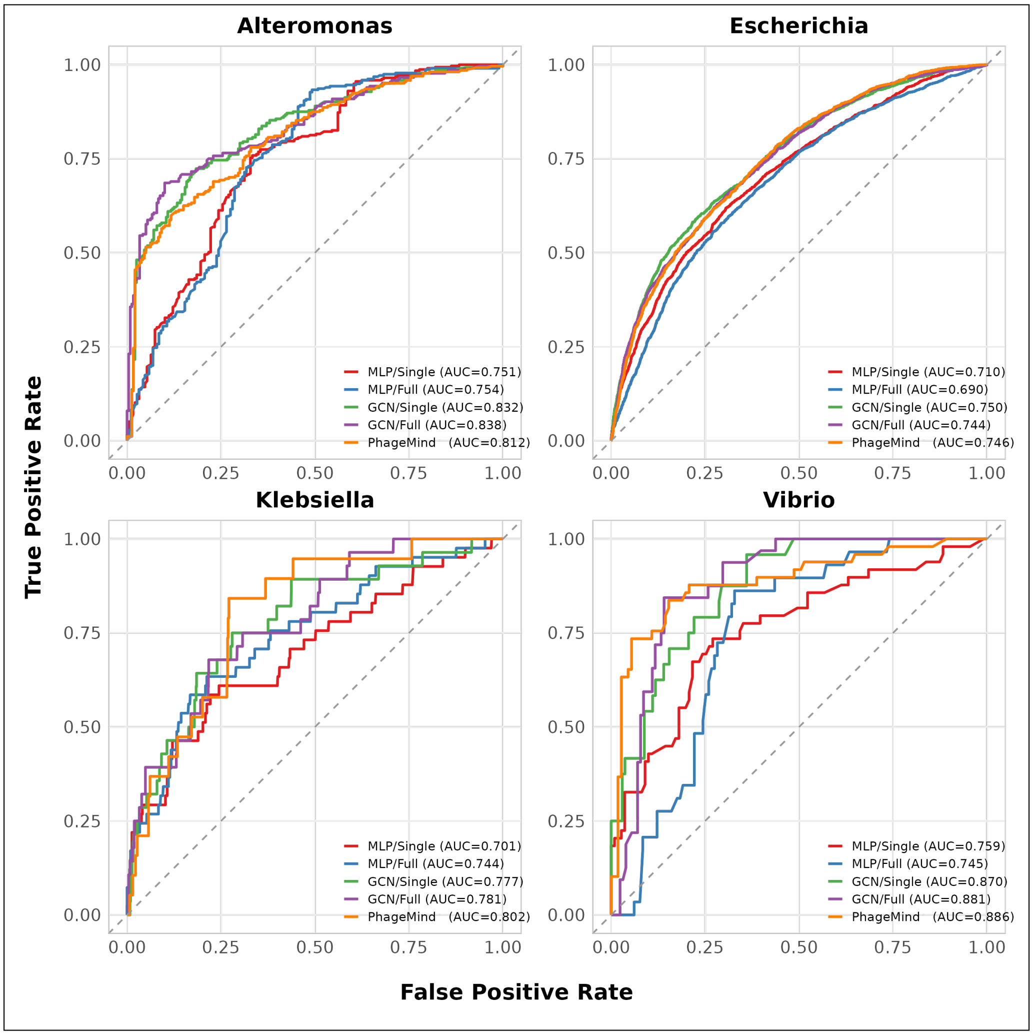

Fig. S2. Best ROC curves across four genera. ROC curves for the single best-performing replicate (highest AUC) from independent training runs for each bacterial genus. Each panel corresponds to one genus and compares PhageMind, GCN, and MLP. GCN/Single and MLP/Single refer to models trained on a single-genus dataset, while GCN/Full and MLP/Full refer to models trained on the combined datasets from all four genera. The diagonal dashed line denotes the no-skill classifier (random guessing). This figure highlights the peak discriminative performance achieved by each method and complements the boxplot analysis of AUC distributions in the main text and Supplementary Figures, showing that PhageMind attains consistently high AUCs and superior sensitivity–specificity trade-offs, with GCN occasionally reaching comparable peak performance and MLP generally exhibiting lower discrimination.

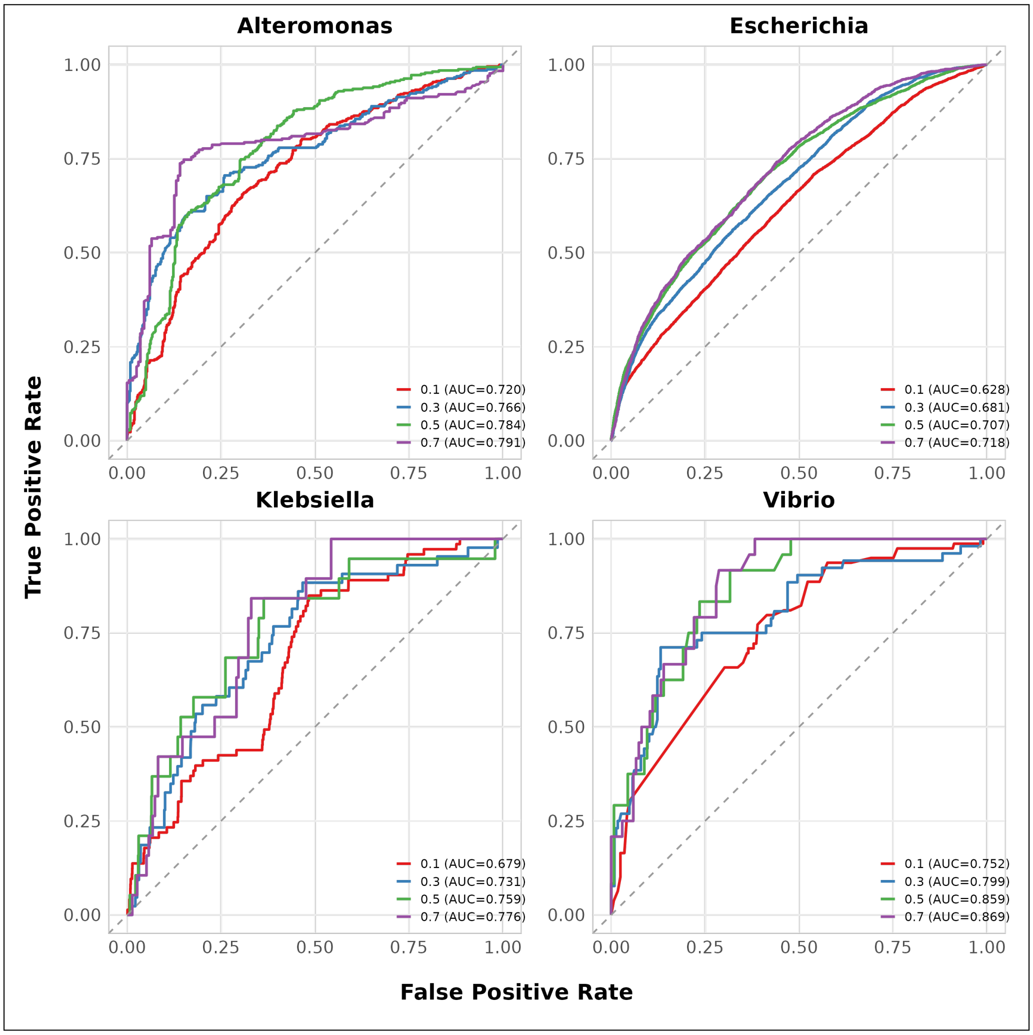

Fig. S3. Few-shot ROC curves across genera under varying fine-tuning fractions. Each subplot displays ROC curves for held-out test data from one genus with colored lines representing classifiers fine-tuned using different fractions of available entities. The x-axis denotes the false positive rate (FPR), and the y-axis denotes the true positive rate (TPR). The diagonal dashed line indicates random performance. As the training fraction decreases (from 0.7 to 0,5, 0.3, and 0.1), ROC curves shift modestly toward the diagonal, reflecting reduced discriminative power. Nonetheless, PhageMind maintains robust classification performance even with limited training data, with curves remaining well above the random baseline. For example, in Escherichia, classifiers trained on just 10% of the data (40 bacteria and 10 phages) still achieve meaningful separation between classes. These results highlight PhageMind's capacity for effective generalization under few-shot conditions, enabling rapid adaptation to novel genera with minimal labeled data.

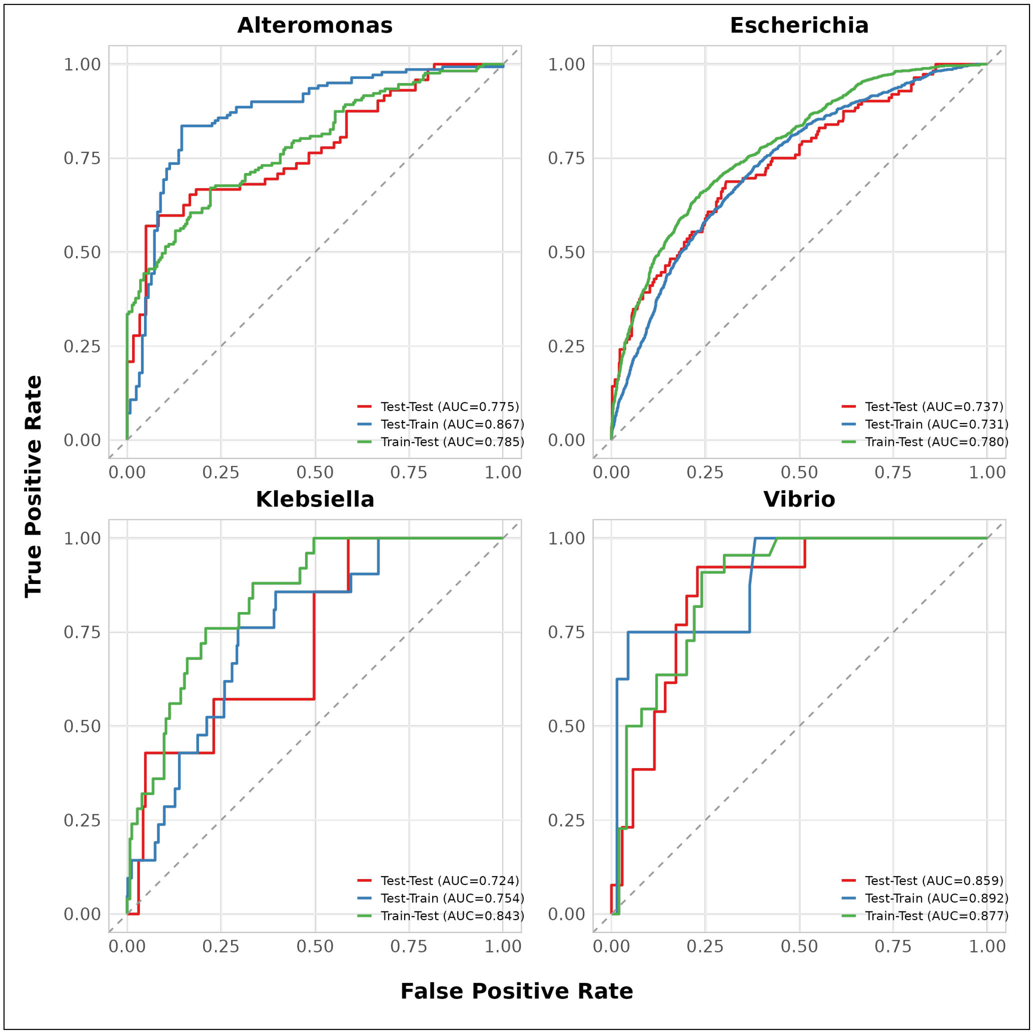

Fig. S4. ROC curves illustrating the performance of PhageMind across the test-training, training-test, and test-test groups for models obtained in Fig. S2. The curves show that while the test-test group represents the most challenging scenario, its AUC values are only modestly lower than the overall results. This indicates that PhageMind is able to capture global patterns across different bacteria and maintain robust predictive ability even when both phage and bacterium strains are unseen.

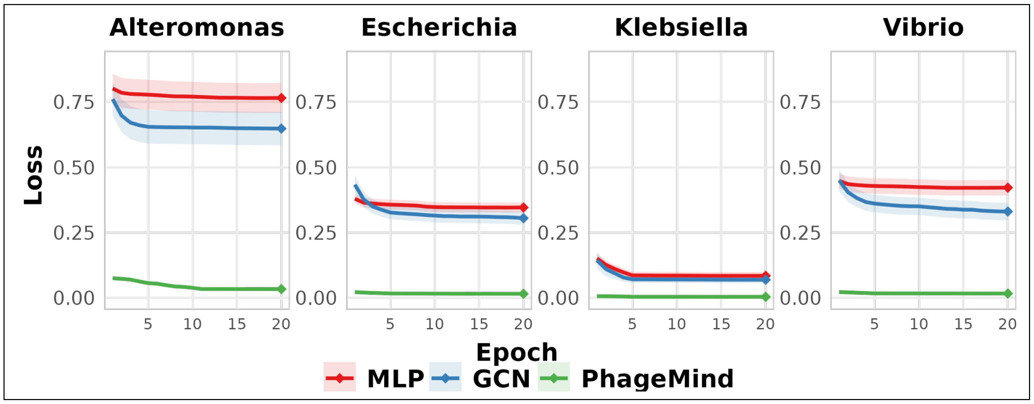

Fig. S5. Dynamics of early training loss for first 20 epochs. The line graph plots the average loss changes across four genera, with shaded areas representing 95% confidence interval. Compared with the rapid decrease observed in the first 5 epochs (Figure 7a), the loss between epochs 5–20 declines more gradually and tends toward stability. In some cases, the MLP baseline shows occasional fluctuations or slight increases in loss, though these are less pronounced and may not be clearly visible in the plot. Our method PhageMind consistently maintains a low loss across epochs, while other methods plateau at higher levels. This pattern indicates that meta-learning avoids local optima by updating initial parameters in a way that better exploits knowledge across genera, thereby achieving a more globally optimal solution.

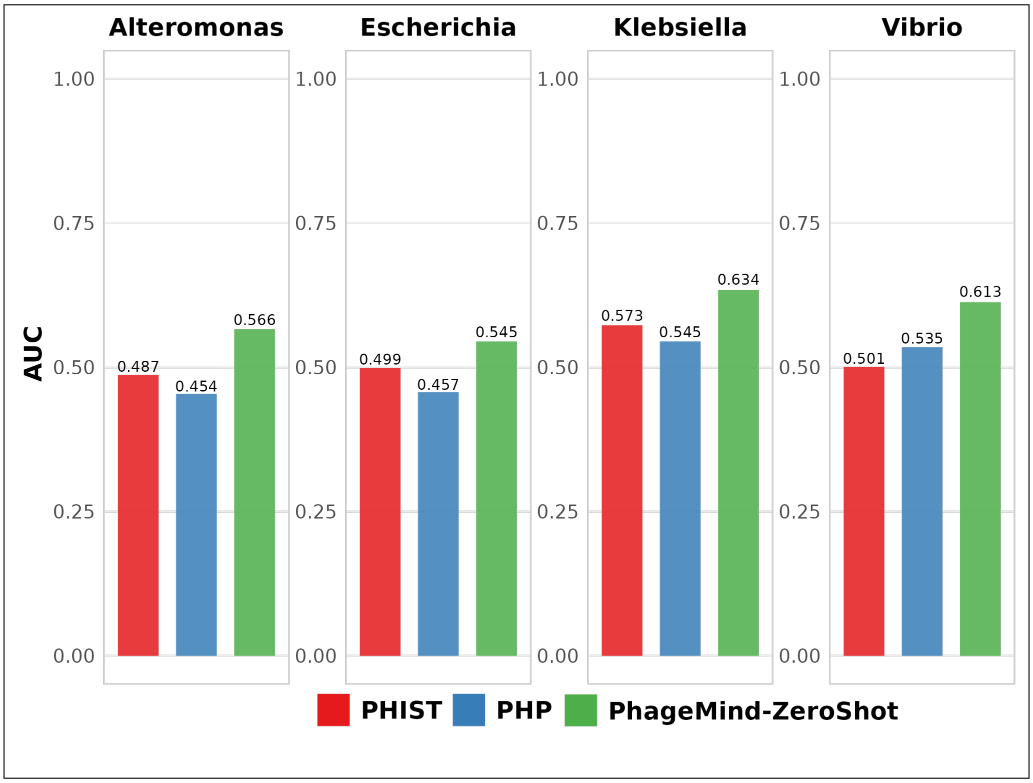

Fig. S6. Comparison of AUC performance between PhageMind (zero-shot), PHP[18], and PHIST[19] across four genera. Each subplot shows grouped bar charts for the three methods. PhageMind-ZeroShot refers to the setting where the model was trained on the other three datasets and directly tested on the unseen dataset, ensuring fair comparison. For PHP, phage-specific scores were normalized to account for heterogeneous ranges and to enable more meaningful AUC calculation. Overall, PhageMind-ZeroShot achieves better performance compared to genome-based methods, highlighting its ability to generalize across unseen datasets.

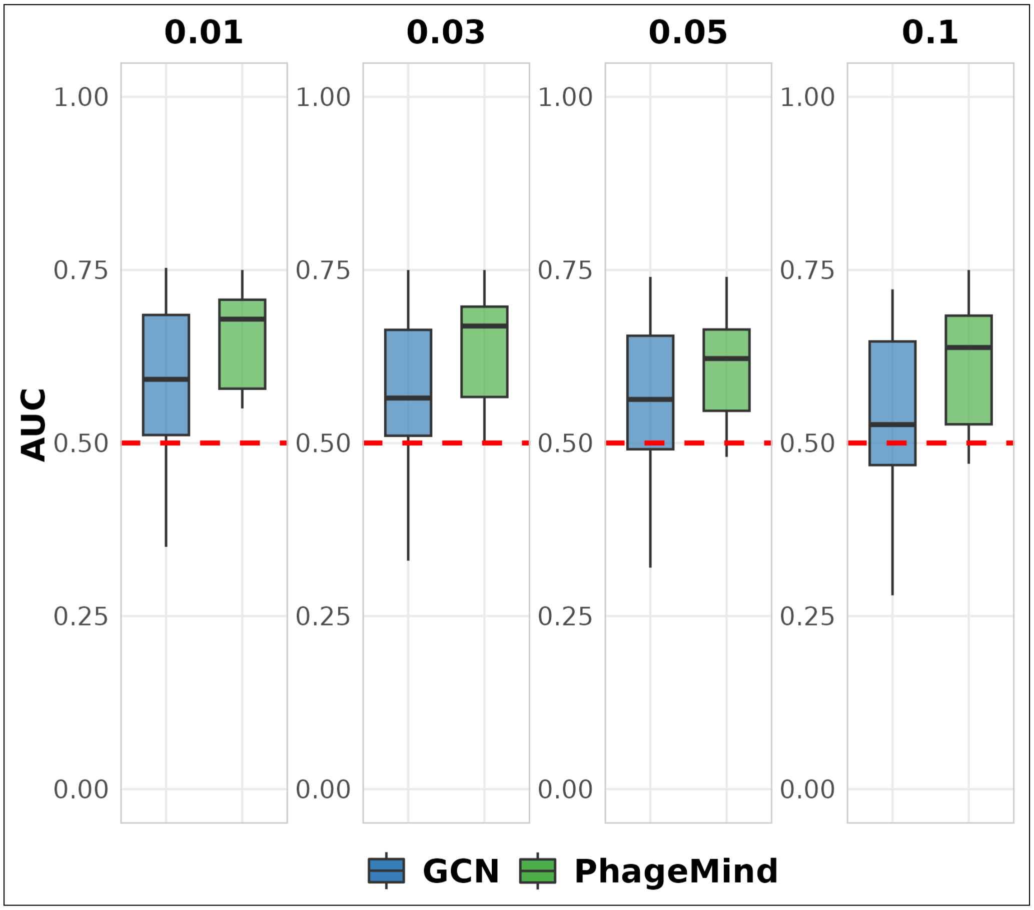

Fig. S7. Box plots of AUC performance for PhageMind (green) and GCN (blue) on the Escherichia dataset with varying levels of added false edges (1%, 3%, 5%, and 10% of total edges). The red dashed line marks the random baseline (AUC = 0.5). PhageMind shows only modest decreases in AUC as noise increases, while GCN exhibits slightly larger declines. In some cases, GCN achieves high AUC values, but its variance is greater. By contrast, PhageMind maintains smaller variance and more stable performance, highlighting its robustness to noisy edges.

REFERENCES

1. B. Liu, A. Furevi, A. V. Perepelov, *et al.*, “Structure and genetics of Escherichia coli O antigens,” FEMS Microbiol. Rev. **44**, 655–683 (2019).
2. M. Steinegger and J. Söding, “MMseqs2 enables sensitive protein sequence searching for the analysis of massive data sets,” Nat. Biotechnol. **35**, 1026–1028 (2017).
3. M. Alonge, L. Lebeigle, M. Kirsche, *et al.*, “Automated assembly scaffolding using RagTag elevates a new tomato system for high-throughput genome editing,” Genome Biol. **23**, 258 (2022).
4. O. I. Naumenko, H. Zheng, Y. Xiong, *et al.*, “Studies on the O-polysaccharide of Escherichia albertii O2 characterized by non-stoichiometric O-acetylation and non-stoichiometric side-chain l-fucosylation,” Carbohydr. Res. **461**, 80–84 (2018).
5. N. Azmuda, M. Z. Rahman, M. Sultana, *et al.*, “Evidence of interspecies O antigen gene cluster transfer between Shigella boydii 15 and Escherichia fergusonii,” APMIS **120**, 959–966 (2012).
6. C.-T. Fang, Y.-J. Shih, C.-M. Cheong, and W.-C. Yi, “Rapid and Accurate Determination of Lipopolysaccharide O-Antigen Types in Klebsiella pneumoniae with a Novel PCR-Based O-Genotyping Method,” J. Clin. Microbiol. **54**, 666–675 (2016).
7. Z. Huang, K. Yu, R. Lan, *et al.*, “Vibrio metschnikovii as an emergent pathogen: analyses of phylogeny and O-antigen and identification of possible virulence characteristics,” Emerg. Microbes & Infect. **12**, 2252522 (2023). PMID: 37616379.
8. A. Aydanian, L. Tang, J. G. Morris, *et al.*, “Genetic Diversity of O-Antigen Biosynthesis Regions in Vibrio cholerae,” Appl. Environ. Microbiol. **77**, 2247–2253 (2011).
9. S. Mustapha, M. M. Ennaji, and N. Cohen, “Vibrio Alginolyticus: An Emerging Pathogen of Foodborne Diseases,” Maejo international journal science technology **2**, 302–309 (2013).
10. D. Oyanedel, Y. Labreuche, M. Bruto, *et al.*, “Vibrio splendidus O-antigen structure: a trade-off between virulence to oysters and resistance to grazers,” Environ. Microbiol. **22**, 4264–4278 (2020).
11. G. Guo, Z. Yang, C. Zhao, *et al.*, “Development of an in silico serotyping database of Vibrio harveyi by identification and comparison of O-antigen encoding loci,” Aquaculture **593**, 741251 (2024).
12. Y. Chen, J. Dai, J. G. Morris, and J. A. Johnson, “Genetic analysis of the capsule polysaccharide (K antigen) and exopolysaccharide genes in pandemic Vibrio parahaemolyticus O3:K6,” BMC Microbiol. **10**, 274 (2010).
13. G. Bouras, R. Nepal, G. Houtak, *et al.*, “Pharokka: a fast scalable bacteriophage annotation tool,” Bioinformatics **39**, btac776 (2022).
14. J. Jumper, R. Evans, A. Pritzel, *et al.*, “Highly accurate protein structure prediction with AlphaFold,” Nature **596**, 583–589 (2021).
15. T. Wei, C. Lu, H. Du, *et al.*, “DeepPBI-KG: a deep learning method for the prediction of phage-bacteria interactions based on key genes,” Briefings Bioinforma. **25**, bbae484 (2024).
16. I. Dubchak, I. Muchnik, S. R. Holbrook, and S. H. Kim, “Prediction of protein folding class using global description of amino acid sequence.” Proc. Natl. Acad. Sci. **92**, 8700–8704 (1995).
17. I. Dubchak, I. Muchnik, C. Mayor, *et al.*, “Recognition of a protein fold in the context of the SCOP classification,” Proteins: Struct. Funct. Bioinforma. **35**, 401–407 (1999).
18. Z.-H. Du, J.-P. Zhong, Y. Liu, and J.-Q. Li, “Prokaryotic virus host prediction with graph contrastive augmentaion,” PLOS Comput. Biol. **19**, 1–19 (2023).
19. A. Zielezinski, S. Deorowicz, and A. Gudyś, “PHIST: fast and accurate prediction of prokaryotic hosts from metagenomic viral sequences,” Bioinformatics **38**, 1447–1449 (2021).
